# Efficacy of repeat hepatectomy versus radiofrequency ablation for recurrent hepatocellular carcinoma: a Systematic Review and meta-analysis

**DOI:** 10.3389/fonc.2025.1559491

**Published:** 2025-03-26

**Authors:** Qingchen Meng, Xiaohang Li, Hongxin Lang

**Affiliations:** ^1^ Department of General Surgery, Graduate School, China Medical University, Shenyang, China; ^2^ Department of Hepatobiliary Surgery, First Affiliated Hospital, China Medical University, Shenyang, China; ^3^ Department of Stem Cells and Regenerative Medicine, Shenyang Key Laboratory for Stem Cells and Regenerative Medicine, Key Laboratory of Cell Biology, Ministry of Public Health, China Medical University, Shenyang, China; ^4^ Key Laboratory of Medical Cell Biology, Ministry of Education, China Medical University, Shenyang, China

**Keywords:** recurrent hepatocellular carcinoma, repeat hepatectomy, radiofrequency ablation, meta-analysis, systematic review

## Abstract

**Background:**

This article compared the efficacy and safety of repeat hepatectomy (RH) and radiofrequency ablation (RFA) for the treatment of recurrent hepatocellular carcinoma (RHCC) from multiple perspectives.

**Methods:**

We systematically searched PubMed, Embase, Web of Science, and CNKI from January 2008 to December 2023. We collected all relevant articles and assessed the quality of the data. We analyzed the data for the primary outcomes of overall survival (OS) and disease-free survival (DFS), and secondary outcomes of postoperative complications, recurrence rate, and survival benefit. Subgroup analyses were performed for tumor diameter, patient origin, and publication date.

**Results:**

A total of 38 studies were included, comprising 5,339 patients. OS was similar in both groups (HR: 0.92, 95% CI: 0.84–1.00, P=0.04, Z=2.02), whereas DFS was better in the RH compared with the RFA group (HR: 0.80, 95% CI: 0.75–0.86, P<0.00001, Z=6.15). The incidence of major complications was lower in the RFA compared with the RH group (OR: 5.06, 95% CI: 3.29–7.81, P<0.00001, Z=7.35), but the postoperative recurrence rate was better in the RH compared with the RFA group. There was no significant difference in postoperative mortality between the two groups, but hospital stay was longer in the RH compared with the RFA group. In subgroup analyses, both OS and DFS were superior in the RH compared with the RFA group among patients with tumors ≤3 cm diameter with no significant difference in the >3 cm-diameter group. There was no significant difference in OS between the two groups among Chinese or non-Chinse patients; however, DFS was higher in the RH compared with the RFA group among Chinese and non-Chinese patients. There was no significant difference in OS between the two groups in studies published pre-2015 or post-2015 group; however, DFS was superior in the RH compared with the RFA group for both pre-2015 and post-2015 studies.

**Conclusion:**

RH may be the first-choice treatment considering the long-term prognosis of patients with RHCC; RFA may be a better alternative in terms of postoperative and economic factors. RH is associated with a better prognosis in patients with tumors ≤3 cm in diameter.

## 1 Introduction

Hepatocellular carcinoma (HCC) is the third leading cause of cancer-related deaths worldwide (1)and the main cause of death from cirrhosis of the liver (2). Despite recent improvements in diagnostic methods and therapeutic options for HCC, its prognosis remains poor, and recurrence is a clinical problem in up to 70% of all patients receiving radical treatment (3). There is still no clear strategy for preventing recurrence, and identifying suitable treatment plans for managing recurrent HCC (RHCC) is thus a high priority. There are various clinical treatment options for RHCC, including salvage liver transplantation, repeat hepatectomy (RH), radiofrequency ablation (RFA), and transarterial chemoembolization (TACE). Among these treatments, salvage liver transplantation is often considered the most effective therapy because it removes both the malignant lesion and the underlying liver disease; however, its implementation has been limited by donor shortages (4), and RH and RFA have thus become the main treatment options for RHCC. The choice between RH and RFA for RHCC remains controversial, and the conclusions of the nine previously published papers varied (5–13). In terms of long-term survival, four papers concluded that RH was superior to RFA (6, 10–12), four concluded that RH and RFA were equivalent in terms of effectiveness (5, 7, 9, 13), and one concluded that RFA was superior to RH under the Milan criteria (8). Despite numerous studies in the past few years, there is still no consensus, possibly because of the small sample sizes. We therefore conducted a meta-analysis of the relevant literature published in the last 15 years to compare the superiority of RH versus RFA for the treatment of RHCC.

## 2 Methods

### 2.1 Search strategy

In this meta-analysis, we systematically searched for articles on the effectiveness of RH and RFA for the treatment of RHCC from January 2008 to December 2023 using the PubMed, Embase, Web of Science, and CKNI databases. A combination of Boolean operators’ Medical Subject Headings (MeSH) terms and non-MeSH terms was used in PubMed. The search results were filtered according to the full text and appropriate articles were selected according to the following inclusion and exclusion criteria: (“Carcinoma,hepatocellular”[MeSH Terms]) OR (Carcinomas,Hepatocellular[All Fields]) OR (Hepatocellular Carcinomas[All Fields]) OR (Liver Cell Carcinoma,Adult[All Fields]) OR (Liver Cancer,Adult[All Fields]) OR (Adult Liver Cancer[All Fields]) OR (Adult Liver Cancers[All Fields]) OR (Cancer,Adult Liver [All Fields]) OR (Cancers,Adult Liver [All Fields]) OR (Liver Cancers,Adult[All Fields]) OR (Liver Cell Carcinoma[All Fields]) OR (Carcinoma,Liver Cell[All Fields]) OR (Carcinomas,Liver Cell[All Fields]) OR (Cell Carcinoma,Liver[All Fields]) OR (Cell Carcinomas,Liver[All Fields]) OR (Liver Cell Carcinomas[All Fields]) OR (Hepatocellular Carcinoma[All Fields]) OR (Hepatoma[All Fields]) OR (Hepatomas[All Fields] AND “Recurrence”[MeSH Terms]) OR (Recurrences[All Fields]) OR (Recrudescence[All Fields]) OR (Relapse[All Fields]) OR (Relapses[All Fields] AND “Radiofrequency Ablation”[MeSH Terms]) OR (Ablation,Radiofrequency[All Fields]) OR (Radio Frequency Ablation[All Fields]) OR (Ablation,Radio Frequency[All Fields]) OR (Radio-Frequency Ablation[All Fields]) OR (Ablation,Radio-Frequency[All Fields] AND “Hepatectomy”[MeSH Terms]) OR (Hepatectomies[All Fields]) OR (Liver Resection[All Fields]) OR (Hepatic Resection[All Fields]) OR (Liver Surgery[All Fields]) OR (Hemihepatectomy[All Fields]). Finally, we also searched relevant cited references in the retrieved articles to identify other eligible articles.

### 2.2 Eligibility criteria

Inclusion criteria: i) Types of articles included randomized controlled trials (RCTs), cohort trials (prospective studies, retrospective studies), and case-control trials; ii). comparison of RHR and RFA (including microwave ablation versus radiofrequency ablation) for the treatment of RHCC must be included in the study, even if there is a third treatment modality, such as TACE; iii) patients diagnosed with RHCC according to the European Association for the Study of the Liver’s diagnostic criteria for HCC (3); iv) first treatment for HCC must be radical (including surgical resection as well as ablation); v) study subjects were patients with first recurrence of HCC; vi) at least 10 patients included in the intervention and control groups; vii) RHCC had no macrovascular invasion visible to the naked eye and no extrahepatic distant metastases; viii) outcomes should include at least one objective assessment (overall survival [OS], disease-free survival [DFS], complications, mortality, recurrence rate, and days of hospitalization; and ix) language of the article restricted to English or Chinese.

Exclusion criteria: i) Other liver tumors (non-HCC); ii) non-first recurrence of HCC (≥2 times); iii) missing or lost postoperative follow-up results; iv) received other treatments prior to receiving RHR or RFA; v) multiple duplicate articles published by the same author with the same data; and vi) articles with no raw data (comments, conference proceedings, letters, replies, reviews, meta-analysis) and non-human experiments.

### 2.3 Data extraction

The following data were extracted from the full text of the included articles: baseline status (first author, year of publication, type of study, number of experimental subjects in the intervention versus control group, sex, age, proportion of hepatitis B surface antigen-positive subjects, number of hepatitis C virus-positive subjects, size of recurrent tumors, number of tumors, Child-Pugh classification, alpha-fetoprotein [AFP], proportion of cirrhosis, duration of follow up, and interval to recurrence), objective outcome indicators (1-/3-/5-year OS, 1-/3-/5-year DFS, complications, days of hospitalization, mortality, postoperative recurrence rate, intraoperative blood loss, and cost of surgery).

### 2.4 Quality assessment

The Newcastle-Ottawa Scale (NOS) is commonly used to assess the quality of included cohort studies (14). The NOS scale consists of three parts: selection, comparability, and outcome. Selection includes representativeness of exposure, representativeness of non-exposed, determination of exposure and outcome not present at start. Comparability includes comparability of most important factors and comparability of other risk factors. Outcome includes assessment of outcome, acceptable length of follow-up, and adequacy of follow-up. The table assesses the treatment of the included articles numerically, with each item potentially scoring 1 point and a total possible score of 9 points. A score ≥7 is considered to indicate a high-quality article and a score <6 is considered a low-quality article. The scores are shown in **Table 1**. (This table was mainly used to evaluate the quality criteria of cohort studies and was not included in the relevant randomized controlled trials).

**Table 1:** Results of quality assessment by Newcastle-Ottawa Scale.

Selection					Comparability			Outcome		Total Score
Study	Representativeness of exposure	Representativeness of non-exposed	Determination of exposure	Outcome not present at start	Comparability on most important factors	Comparability on other risk factors	Assessment of outcome	Acceptable length of follow up	Adequacy follow up	
Chan	☆	☆	☆	☆	☆		☆	☆	☆	8
Chen K	☆	☆	☆	☆			☆	☆	☆	7
Chen S	☆	☆	☆	☆	☆	☆	☆	☆	☆	9
Chua	☆	☆	☆	☆			☆	☆	☆	7
Duan	☆	☆	☆	☆	☆	☆	☆	☆	☆	9
Eisele			☆	☆			☆	☆	☆	5
Feng	☆	☆	☆	☆			☆	☆	☆	7
Hirokawa	☆	☆	☆	☆			☆	☆	☆	7
Ho	☆	☆	☆	☆			☆	☆	☆	7
Huang	☆	☆	☆	☆			☆	☆	☆	7
Imai	☆	☆	☆	☆			☆	☆	☆	7
Joliat	☆	☆	☆	☆			☆	☆	☆	7
Karabulut	☆	☆	☆	☆			☆	☆	☆	7
Kawano Y	☆	☆	☆	☆			☆	☆	☆	7
Kim	☆	☆	☆	☆			☆	☆	☆	7
Liang	☆	☆	☆	☆	☆	☆	☆	☆	☆	9
Liang	☆	☆	☆	☆	☆	☆	☆	☆	☆	9
Lu	☆	☆	☆	☆			☆	☆	☆	7
Matsumoto	☆	☆	☆	☆	☆		☆	☆	☆	8
Peng Z	☆	☆	☆	☆			☆	☆	☆	7
Ren	☆	☆	☆	☆		☆	☆	☆	☆	8
Satio	☆	☆	☆	☆			☆	☆	☆	7
Shen	☆	☆	☆	☆	☆	☆	☆	☆	☆	9
Song	☆	☆	☆	☆		☆	☆	☆	☆	8
Sun	☆	☆	☆	☆	☆	☆	☆	☆	☆	9
Umeda			☆	☆			☆	☆	☆	5
Wang	☆	☆	☆	☆			☆	☆	☆	7
Wang	☆	☆	☆	☆			☆	☆	☆	7
Wei	☆	☆	☆	☆			☆	☆	☆	7
Xiao	☆	☆	☆	☆			☆	☆	☆	7
Yan K	☆	☆	☆	☆	☆	☆	☆	☆	☆	9
Yin	☆	☆	☆	☆	☆	☆	☆	☆	☆	9
Zhang H	☆	☆	☆	☆			☆	☆	☆	7
Zhang T	☆	☆	☆	☆	☆	☆	☆	☆	☆	9
Zhang	☆	☆	☆	☆		☆	☆	☆	☆	8
Zhong	☆	☆	☆	☆			☆	☆	☆	7

☆ means "Yes".

### 2.5 Outcomes and definitions

We categorized the major objective outcome indicators into primary and secondary outcomes. The primary outcomes were OS (1-/3-/5-year) and DFS (1-/3-/5-year). OS was defined as the time from the start of randomization (or the start of treatment in a single-arm trial) to death from any cause, and from the start of the first recurrence of HCC in patients undergoing RH or RFA to the time of death or loss to follow-up. DFS was defined as the time from the start of randomization (or the start of treatment in a single-arm trial) to disease recurrence or death from any cause, and from the start of the first recurrence of HCC to the second recurrence of the disease or death from the disease. The secondary outcomes included hospital days, postoperative complications, postoperative recurrence rate, and mortality rate. Length of hospital stay was defined as the time from the beginning of the patient’s admission to hospital with RHCC for RH or RFA until the time of cure and discharge from the hospital. Complications were categorized using the Clavien-Dindo scale (15). Postoperative recurrence rate was defined as the proportion of patients with RHCC who had a second recurrence after RH or RFA during the effective follow-up period. Mortality was defined as the proportion of patients with RHCC who died from the disease after RH or RFA.

### 2.6 Statistical analysis and heterogeneity testing

This meta-analysis was performed using Review Manager version 5.3 software provided by the Cochrane Collaboration (16). A forest plot was generated to visualize the results. The two main outcome metrics of 1-, 3-, and 5-year OS and DFS, respectively, were extracted directly from the original literature, or for the few cases where data were not provided in the paper, we extracted survival data via the Kaplan-Meier survival curves provided in the text using the method provided by Tierney et al. (17). Because this meta-analysis included studies that used propensity score matching, the extracted survival data comprised the original data prior to propensity score matching. Hazard ratios (HRs) with 95% confidence intervals (CIs) were calculated for the analysis of outcomes for OS and DFS, days of hospitalization were analyzed using standardized mean differences (SMD), and odds ratios (ORs) with 95% CIs were used to compare the secondary outcomes of major complications, postoperative recurrence rates, and mortality. Finally, an effects model (fixed/randomized) was used to generate overall effect sizes and 95% CIs to analyze the differences between the two groups. We also explored the heterogeneity between the outcome indicators using the χ^2^ test and I2 statistic, A P value <0.05 was considered statistically significant. An I2 <50% suggested that the difference between the two groups was too small to be compared using a fixed-effect model, and an I2 >50% was considered to indicate significant variability, and a random-effects model was used. The overall effect pooled for each group was ultimately determined by Z-test, with P<0.05 indicating a significant result.

### 2.7 Publication bias

We assessed the likelihood of publication bias using funnel plots (18): asymmetric, plots indicated publication bias, which could be related to publication bias or to factors such as clinical or methodological heterogeneity among studies. We therefore also assessed the presence of publication bias using the trim and fill method, Egger regression test, and Begg test (19).

## 3 Results

### 3.1 Study selection and clinical baseline characteristics

**Figure 1:**
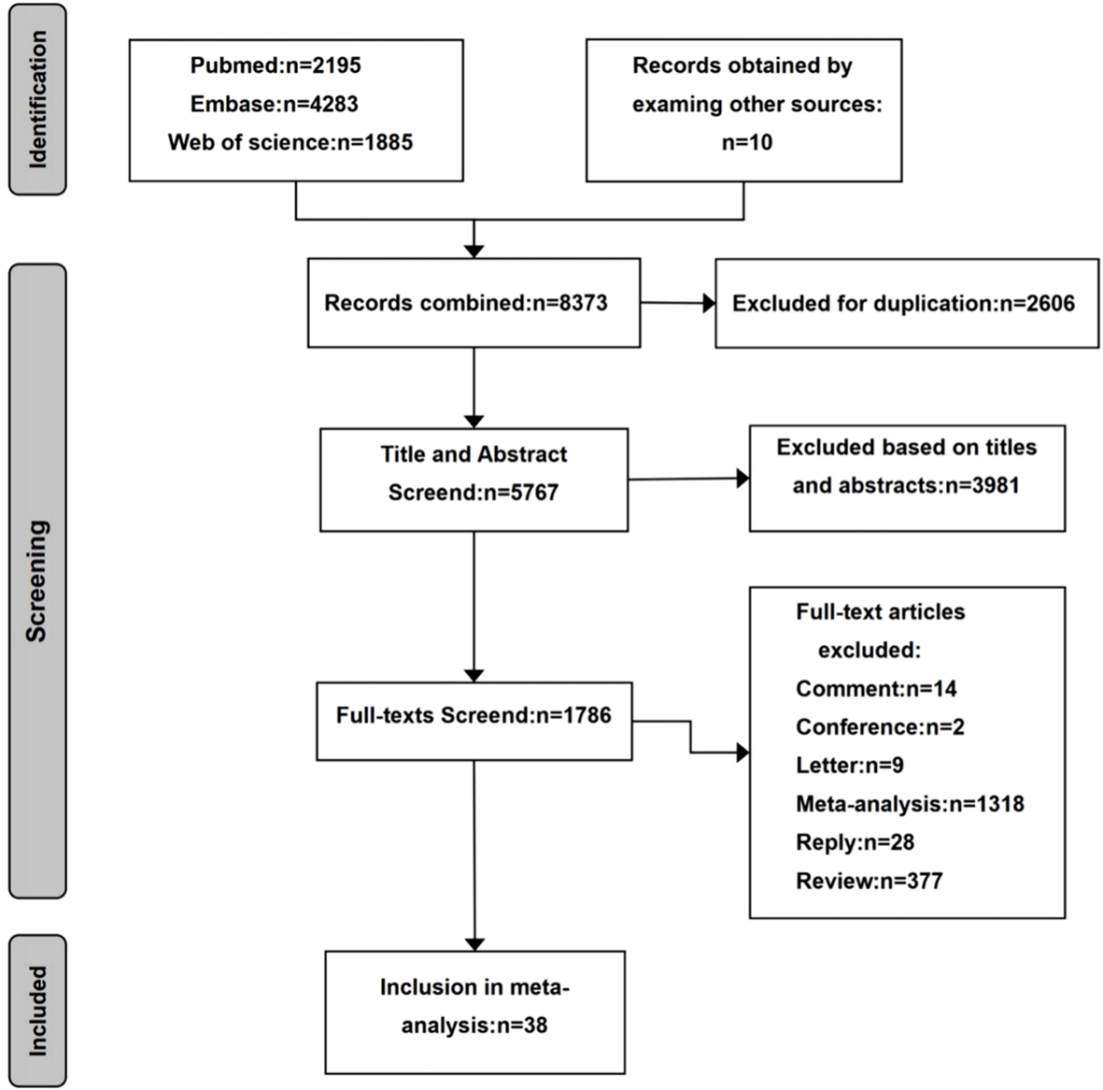
Flow chart for article screening.

In this meta-analysis, we retrieved a total of 8,373 relevant articles through the major databases, which resulted in 5,767 articles after excluding 2,606 duplicates. Thirty-eight eligible articles were finally included after screening the article contents (title, abstract, article type) (20–23). The search process is presented in **Figure 1**.

The 38 studies were published from 2008 to 2023 and included a total of 5,339 patients (RH group: 2,254; RFA group: 3,085) Twenty-six groups of patients were from China (including Taiwan and Hong Kong) (9, 20–22, 24–45), six from Japan (46–51), two from Korea (52, 53), one from Singapore (23), one from USA (54), one from Germany (55), and one from Switzerland (56). There were two RCTs (30, 38)and most of the rest were cohort studies. Thirty-five articles recorded OS rates (20, 23), 25 recorded DFS rates (9, 20, 22, 23, 25, 27, 30–33, 35, 36, 38, 40, 41, 43, 45–47, 49, 50, 52–55), 19 reported the associated complications (9, 20, 22, 25, 29–32, 34–36, 38, 40, 41, 45, 47, 53, 55, 56), 15 reported postoperative recurrence rates (20, 22, 28, 30, 31, 34, 35, 38, 41, 45, 47, 49, 52–54), nine recorded information on the number of days of hospitalization (22–24, 30, 32, 34, 36, 47, 54), and seven reported postoperative mortality (20, 23, 24, 32, 43, 47, 54). Notably, 13 publications reported the initial or secondary surgical approach (open or laparoscopic) for patients with RHCC (20, 24, 25, 28, 30, 32, 34, 35, 38, 41, 45, 53, 55), which included both RH and RFA, while 14 papers documented the type of RH (anatomical and non-anatomical resection) (9, 22, 25, 30–32, 37, 39, 40, 46, 47, 49, 51, 55).

The clinical baseline information for the included studies is presented in **Table 2**. There was no significant difference between the RH and RFA groups in terms of sex, but patients in the RFA group were generally older than those in the RH group and had more and larger recurrent nodules, whereas the RH group was slightly superior to the RFA group in terms of hepatic functional reserve.

**Table 2:** Baseline characteristics of included studies.

First author	Year	Country	Treatment	Number	Research type	Gender (M,%)	Age	HBsAg (+)%	HCV (+)	Recurrent Tumor size (cm)	Tumor number (%)	Child-Pugh class (A/B/C)	AFP (ng/ml)%	Liver Cirrhosis (%)	Follow-up time (month)	Time interval-recurrence (month %)
Liang	2008	China	RHR RFA	44 66	Retrospective	88.63 81.82	48 54	93.18 90.91	NR NR	≤3/>3:59.1/40.9 ≤3/>3:66.7/33.3	1/2/3:77.3/20.4/2.3 1/2/3:77.7/21.2/6.1	44/0/0 64/2/0	<400:68.2 <400:78.8	NR NR	33.5 ± 24.1 21.1 ± 19.1	≤12/>12:40.9/59.1 ≤12/>12:37.9/62.1
Ren	2008	China	RHR RFA	145 68	Retrospective	87.59 94.12	51 52	87.59 83.82	1 1	2.0 2.0	S/M:86.9/13.1 S/M:77.9/22.1	145/0/0 68/0/0	NR NR	NR NR	23 (3-88) 23 (3-88)	≤24/>24:48.97/51.03 ≤24/>24:54.41/45.59
Shen	2008	China	RHR RFA	31 45	Retrospective	80.65 82.22	51.3 ± 2.2 56.8 ± 1.7	93.5 88.9	1 3	3.2 ± 0.2 2.9 ± 0.1	S/M:71.0/29.0 S/M:55.5/44.5	29/2/0 37/8/0	≤20/>20:58.1/41.9 ≤20/>20:55.6/44.4	NR NR	NR NR	NR NR
Kawano Y	2009	Japan	RHR RFA	13 33	Retrospective	NR NR	NR NR	NR NR	NR NR	NR NR	NR NR	NR NR	NR NR	NR NR	NR NR	NR NR
Umeda	2011	Japan	RHR RFA	29 58	Retrospective	NR NR	≥65 (16) ≥65 (37)	27.6 19	19 41	3.2 ± 0.57 2.1 ± 0.3	S/M:62.1/37.9 S/M:58.1/41.2	29/0/0 51/7/0	≥100:16.7 ≥100:5.6	NR NR	48 48	≤24/>24:51.72/48.28 ≤24/>24:62.07/37.93
Karabulut	2011	USA	RHR RFA	92 92	Retrospective	64 80	68 64	NR NR	45 50	5.3 3.1	1.2 ± 0.1 1.6 ± 0.1	43/0/0 50/29/13	1,073 ± 492 1,236 ± 534	46.7 100	NR NR	NR NR
Duan	2011	China	RHR RFA	35 26	Retrospective	97.14 84.62	48.46 ± 8.49 51.96 ± 5.64	NR NR	NR NR	7.34 ± 3.16 5.59 ± 3.40	1:100 1:100	NA/NA/NA NA/NA/NA	NR NR	NR NR	NR NR	41.32 ± 33.9 30.11 ± 32.5
Hirokawa	2011	Japan	RHR RFA	10 21	Retrospective	80.00 80.95	69 67	50 62	NR NR	1.9 ± 0.7 1.7 ± 0.6	S/M:70.0/30.0 S/M:76.2/23.8	NR/2/NR NR/3/NR	NR NR	30 38	NR NR	22.8 (8.8-120) 7.6 (2.9-64.2)
Chan	2012	China	RHR RFA	29 45	Retrospective	NR NR	52 (38-79) 59 (36-80)	89.66 88.89	3 5	2.1 (0.8-5.5) 2.2 (0.8-6.0)	S/M:72.4/27.6 S/M:64.4/35.6	29/0/0 40/5/0	64 (2-167,138) 90 (1-197,122)	86 89	44.9 (8.3-112) 44.9 (8.3-112)	12.2 (1.8-84.3) 8.7 (1.0-88.5)
Ho	2012	China	RHR RFA	54 55	Retrospective	74.1 78	56.3 ± 12.3 61.0 ± 11.1	72.2 54	17 25	2.9 ± 1.8 2.3 ± 1.9	≤3/>3:96.0/4.0 ≤3/>3:90.0/10.0	51/2/1 50/0/0	NR NR	48.1 56	32 ± 19.75 27 ± 24	23.9 ± 15.8 20 ± 29
Eisele	2013	Germany	RHR RFA	27 27	Retrospective	55.6 74.1	60 ± 17 68 ± 7	NR NR	8 11	4.0 ± 2.3 2.8 ± 1.1	S/M:59/41 S/M:56/44	27/0/0 19/8/0	NR NR	37 82	34 21	39 ± 27 21 ± 17
Zhang T	2014	China	RHR RFA	27 39	Retrospective	NR NR	47 52	96.3 94.9	NR NR	3.2 ± 1.1 2.7 ± 1.1	S/M:92.6/7.4 S/M:94.9/5.1	27/0/0 37/2/0	≤100/>100:59.3/40.7 ≤100/>100:79.5/20.5	NR NR	32 (9-118) 28 (2-79)	36 ± 19 30 ± 20
Imai	2014	Japan	RHR RFA	23 27	Retrospective	78.3 66.7	67.2 ± 9.5 71.0 ± 7.7	26.1 11.1	14 22	3.17 ± 2.38 1.78 ± 0.5	2.3 ± 1.4 1.2 ± 0.6	23/0/0 21/6/0	508.2 ± 1575 922.8 ± 4464	NR NR	28.7 ± 29.3 35.3 ± 25.4	19.0 ± 18.0 21.2 ± 26.1
Song	2015	Korea	RHR RFA	39 178	Retrospective	79 81.5	52.5 ± 9.8 55.4 ± 10.6	92 83.7	1 12	2.2 ± 1.1 1.7 ± 0.6	S/M:82/18 S/M:83/17	39/0/0 172/6/0	≤200/>200:84.6/15.4 ≤200/>200:92.1/7.9	59 73	36.3 (0.8-126.6) 44.7 (5.6-139.8)	20.9 (3.1-136.3) 18.0 (3.1-118.4)
Wang	2015	China	RHR RFA	128 162	Cohort	88.3 91.4	50.2 ± 10.1 52.7 ± 10.9	93 87.7	NR NR	2.4 ± 0.9 2.3 ± 0.7	S/M:69.5/30.5 S/M:66.0/34.0	NR/NR/NR NR/NR/NR	≤20/>20:56.2/43.8 ≤20/>20:52.5/47.5	51.6 NR	NR NR	15.1 ± 10.3 14.1 ± 10.5
Zhang	2015	China	RHR RFA	25 23	Retrospective	88	50.54 ± 10.848	88	NR	2.78 ± 1.26	S/M:78/22	49/1/0	≤20/>20:58/42	NR	42.850 ± 22.282	≤12/>12:44/56
Huang	2015	China	RHR RFA	15 11	Cohort	60 72.7	≤50/>50:6/9 ≤50/>50:6/5	80 81.8	1 0	2.21 ± 1.07 2.68 ± 1.31	S/M:80/20 S/M:63.6/36.4	14/1/0 5/6/0	≤400/>400:53.3/46.7 ≤400/>400:54.5/45.5	NR NR	NR NR	NR NR
Sun	2017	China	RHR RFA	43 57	Retrospective	79 67	63 (37-84) 65 (31-84)	49 56	20 22	1.9 (0.8-3.0) 1.8 (1.0-3.0)	S/M:95/5 S/M:96/4	35/1/7 50/0/7	23 (3-290) 67 (2-817)	83 88	53 54	26 (4-126) 14 (1-86)
Joliat	2017	Switzerland	RHR RFA	10 18	Retrospective	80 72.2	68 (54-75) 64 (62-68)	NA NA	NR NR	NR NR	NR NR	5/0/NR 13/3/NR	5 (2-16846) 6 (2-129)	50 88.9	NR NR	NR NR
Chen S	2018	China	RHR RFA	48 57	Retrospective	NR NR	73.5 73.7	79.2 78.9	3 4	2.6 ± 1.135 2.5 ± 1.2	S/M:58.3/41.7 S/M:52.6/47.4	NA/NA/NA NA/NA/NA	≤200/>200:45.8/54.2 ≤200/>200:52.6/47.4	85.4 86.0	36.9 (2-78) 37.3 (2-78)	NR NR
Yin	2019	China	RHR RFA	57 51	Retrospective	71.93 60.78	57 60	92.98 94.12	NR NR	3.2 2.6	S/M:91.23/8.77 S/M:94.12/5.88	55/2/0 46/5/0	167.97 266.32	68.42 58.82	35 (6-60) 37 (7-60)	29 24
Xiao	2019	China	RHR RFA	11 24	Retrospective	90.9 75	≤60 (72.7%) ≤60 (79.2%)	100 87.5	0 0	≤5/>5:72.7/27.3 ≤5/>5:95.8/4.2	S/M:45.5/54.5 S/M:45.8/54.2	11/0/0 24/0/0	≤200/>200:54.5/45.5 ≤200/>200:66.7/33.3	NR NR	NR NR	≤12/>12:72.7/27.3 ≤12/>12:95.8/4.2
Chen K	2019	China	RHR RFA	77 82	Retrospective	84.4 87.8	≤60 (67%) ≤60 (61%)	90.9 97.6	NR NR	≤3: (39%) ≤3: (77%)	NR NR	76/1/0 77/5/0	≤400/>400:66.2/33.8 ≤400/>400:92.7/7.3	74 61	57 (2-168) 51 (4-111)	20 (2-171) 9 (1-75)
Liu JL	2019	China	RHR RFA	39 41	RCT	97.4 90.2	50.0 48.9	94.9 90.2	NR NR	2.09 ± 0.68 1.82 ± 0.82	S/M:94.9/5.1 S/M:95.1/4.9	38/1/0 39/2/0	437.108 ± 1221.208 1087.159 ± 6272.428	94.9 95.1	24 24	33.4 21.9
Lu	2020	China	RHR RFA	138 194	Retrospective	89.9 88.7	50.1 ± 10.9 52.9 ± 11.8	91.3 88.7	2.2 3.1	2.8 ± 1.9 1.9 ± 0.9	S/M:81.2/18.8 S/M:83.5/16.5	138/0/0 194/0/0	≤20/>20:68.8/31.2 ≤20/>20:63.4/36.6	69.6 69.1	NR NR	≤24/>24:39.1/60.9 ≤24/>24:65.5/34.5
Xia	2020	China	RHR RFA	120 120	RCT	89.2 90.8	52.4 (25.7-60.5) 53.5 (28.0-59.9)	NR NR	NR NR	2.9 (1.0-5.0) 2.7 (1.0-4.8)	S/M:80.0/20.0 S/M:78.3/21.7	120/0/0 120/0/0	NR NR	41.7 45.8	44.3 (4.3-90.6) 44.3 (4.3-90.6)	29.5 (5.0-79.7) 26.3 (4.6-61.6)
Feng	2020	China	RHR RFA	48 48	Retrospective	84.8 90.1	56.0 57.9	91.9 90.0	0 4	3.0 (2.5-4.0) 2.2 (1.5-3.0)	S/M:75.8/24.2 S/M:63.4/36.6	96/3/0 182/9/0	NR NR	60.6 66.0	NR NR	>12:79.8 >12:55.5
Kim	2020	Korea	RHR RFA	45 171	Retrospective	91.1 80.1	53 (19-72) 56 (24-80)	77.8 81.9	0 8	3.6 (1.0-21.0) 3.5 (1.0-15.9)	S/M:100/0 S/M:99.4/0.6	NR/NR/NR NR/NR/NR	11.3 (1.0-13509.8) 4.9 (1.0-3199.0)	NR NR	64 (4-113) 60 (6-115)	22 (2-63) 18 (1-85)
Satio	2020	Japan	RHR RFA	17 26	Retrospective	NR NR	NR NR	NR NR	NR NR	NR NR	NR NR	NR/NR/NR NR/NR/NR	NR NR	NR NR	NR NR	NR NR
Yan K	2020	China	RHR RFA	34 22	Retrospective	58.8 72.7	67.7 ± 4.7 68.4 ± 6.6	47.1 59.1	NR NR	3.8 ± 0.7 3.9 ± 0.6	S/M:73.5/26.5 S/M:68.2/31.8	28/6/0 19/3/0	NR NR	41.1 50.0	NR NR	11.7 ± 2.0 11.4 ± 2.3
Chua	2021	Singapore	RHR RFA	92 127	Retrospective	85.9 81.9	60 63	77.1 64.2	1 16	3.0 (2.2-4.0) 3.2 (2.2-4.5)	S/M:94.6/5.4 S/M:72.4/27.6	89/3/0 115/12/0	17 (4-174) 11 (5-58)	52.2 69	NR NR	26.1 (9.1-126.5) 16.2 (7.8-78.0)
Wei	2021	China	RHR RFA	80 46	Retrospective	NR NR	≤45/>45:28.6/71.4 ≤45/>45:14.3/85.7	NR NR	NR NR	<3/3-5:94.3/5.7 <3/3-5:91.4/8.6	S/M:85.7/14.3 S/M:68.6/31.4	80/0/0 46/0/0	NR NR	NR NR	31.0 (7.0-61.0) 31.0 (7.0-61.0)	≤12/>12:54.3/45.7 ≤12/>12:60.0/40.0
Zhong	2021	China	RHR RFA	307 540	Retrospective	79.8 89.3	<60/≥60:217/90 <60/≥60:356/184	85.3 85.0	NR NR	<3/≥3:44.0/56.0 <3/≥3:78.7/21.3	S/M:74.6/25.4 S/M:75.6/24.4	300/7/0 523/17/0	NR NR	58.6 56.3	54 (1-178) 49.3 (1-156)	≤12/>12:26.1/73.9 ≤12/>12:46.9/53.1
Matsumoto	2021	Japan	RHR RFA	23 11	Retrospective	87.0 100	66 67	30.4 18.2	9 6	1.8 (1.0-2.6) 1.8 (0.7-2.5)	S/M:69.6/30.4 S/M:72.7/27.3	20/3/0 8/3/0	NR NR	69.6 54.5	43.2 (1.2-150) 43.2 (1.2-150)	≤36/>36:4.3/95.7 ≤36/>36:36.4/63.6
Wang	2023	China	RHR RFA	79 189	Retrospective	89.9 89.4	47.00 (40.00-55.00) 53.00 (43.00-61.00)	96.2 92.1	2 5	2.20 (1.40-2.90) 1.40 (1.20-1.90)	S/M:86.1/13.9 S/M:91.0/9.0	75/NR/NR 173/NR/NR	6.60 (2.58-67.27) 7.53 (3.00-68.90)	73.4 77.2	41.53 (2.4-129.47) 40.46 (1.83-119.57)	40 (50.6%) 134 (70.9%)
Peng Z	2018	China	RHR RFA	79 107	Retrospective	84.8 88.8	55.00 (18.00-75.00) 57.00 (19.00-75.00)	91.1 91.6	NR NR	≤3/>3:60.8/39.2 ≤3/>3:68.2/31.8	1/2/3:74.7/16.5/8.9 1/2/3:70.1/22.4/7.5	NR/NR/NR NR/NR/NR	≤200/>200:62.0/38.0 ≤200/>200:50.5/49.5	NR NR	NR NR	≤12/>12:58.2/41.8 ≤12/>12:53.3/46.7
Liang	2011	China	RHR RFA	72 79	Retrospective	90.3 87.3	55 ± 11 49 ± 12	95.8 92.4	NR NR	2.16 ± 0.10 2.46 ± 0.09	NR NR	70/2/0 73/6/0	≤25/>25:25.0/75.0 ≤25>25:17.7/82.3	NR NR	NR NR	≤12/>12:54.2/45.8 ≤12/>12:46.8/53.2
Zhang H	2013	China	RHR RFA	69 99	Retrospective	NR NR	NR NR	61 76	NR NR	<3/3-5/>5:47.8/42.0/10.1 <3/3-5/>5:56.6/37.4/6.1	NR NR	54/15/0 71/28/0	NR NR	NR NR	NR NR	15 14

### 3.2 Primary outcomes

#### 3.2.1 OS

**Figure 2:**
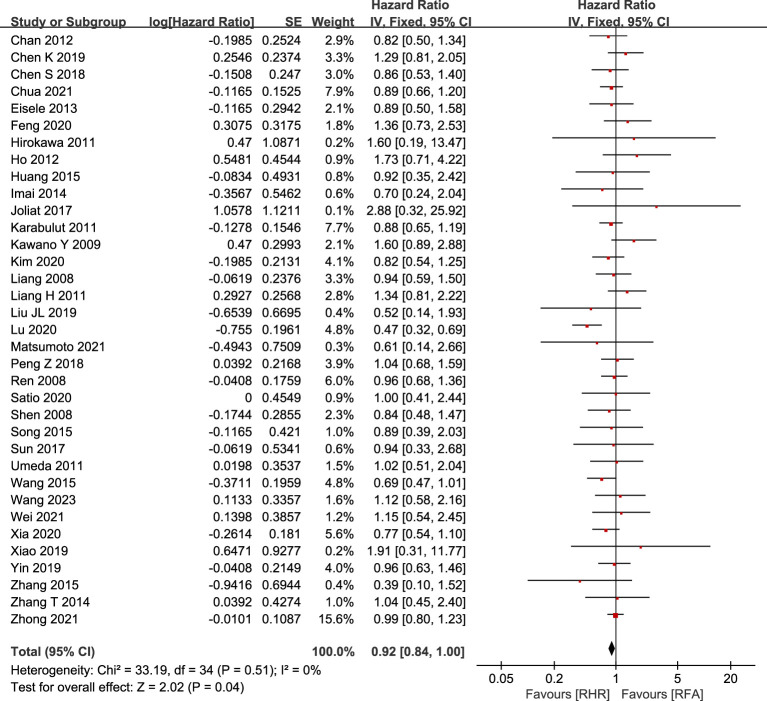
Forest plot for the comparison of hazard ratios and 95% confidence intervals (CI) for overall survival (OS).

**Figure 3:**
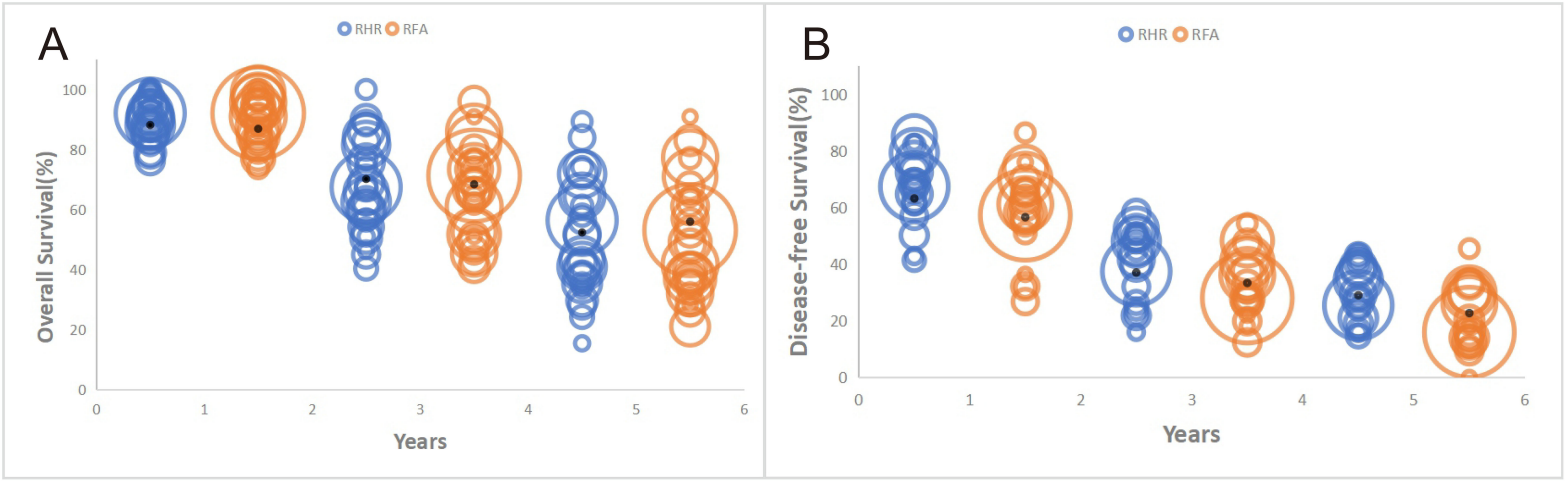
Bubble plots of 1-,3-, and 5-year survival of patients with recurrent hepatocellular carcinoma after RH or RFA. **(A)** Overall Survival, **(B)** Disease-Free Survival.

Relevant OS information was extracted from 35 studies (5,054 patients; RH group: 2,116; RFA group: 2,938). There was no significant difference in OS between the two groups (HR:0.92, 95% CI: 0.84–1.00, P=0.04, Z=2.02) (**Figure 2**), and no heterogeneity in the analysis of OS as a whole (I2 = 0%, P=0.51). A total of eight studies documented median OS (9, 23, 25, 33, 38, 45, 55, 56), which ranged from 32.22 to 85.50 months in the RH group and from 27.25 to 77 months in the RFA group. We also analyzed the 1-, 3-, and 5-year OS rates (**Table 3**). The 28 included studies showed no significant difference between the RH and RFA groups in terms of 1-year OS (OR: 0.91, 95% CI: 0.74–0.92, p=0.37, Z=0.9 and I2 = 3%), 3-year OS (OR: 1.13, 95% CI: 0.99–1.29, P=0.07, Z=1.82, I2 = 38%), or 5-year OS (OR: 1.05, 95% CI: 0.81–1.34, P=0.72, Z=0.36, I2 = 70%). The results of this analysis are shown in bubble plots in **Figure 3A**.

**Table 3:** Subgroup Analysis of Overall Survival and Disease Free Survival.

Subgroup	NO. of Studies	RHR	RFA	Total	Statistical Method	Effect Estimate	Z	I^2^	P
OS									
1-year	28	1871	2530	4401	Odds Ratio (M-H, Fixed, 95% CI)	0.91 [0.74, 1.12]	0.9	3%	p=0.37
3-year	28	1914	2689	4603	Odds Ratio (M-H, Fixed, 95% CI)	1.13 [0.99, 1.29]	1.82	38%	p=0.07
5-year	29	1978	2636	4614	Odds Ratio (M-H, Fixed, 95% CI)	1.05 [0.81, 1.34]	0.36	70%	p=0.72
DFS									
1-year	19	1270	1702	2972	Odds Ratio (M-H, Fixed, 95% CI)	1.53 [1.30, 1.80]	5.18	45%	p<0.00001
3-year	19	1276	1832	3108	Odds Ratio (M-H, Fixed, 95% CI)	1.47 [1.25, 1.71]	4.81	0%	p<0.00001
5-year	19	1323	1753	3076	Odds Ratio (M-H, Fixed, 95% CI)	1.64 [1.38, 1.95]	5.6	28%	p<0.00001

#### 3.2.2 DFS

**Figure 4:**
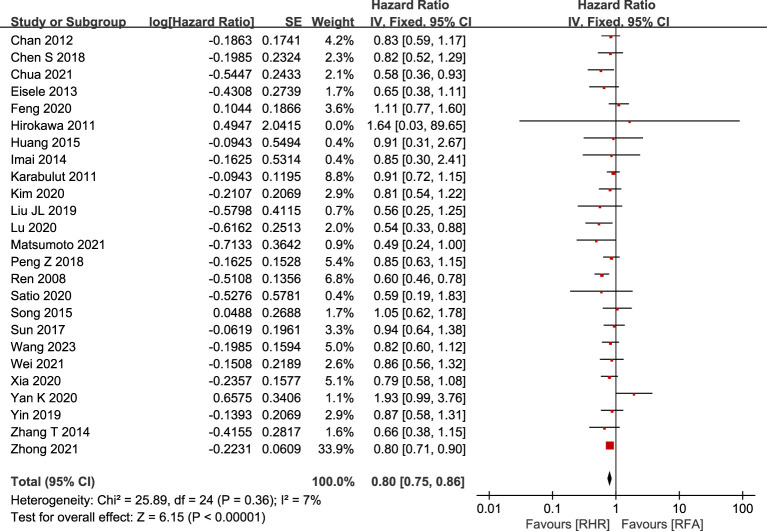
Forest plot for comparison of hazard ratios and 95%CIs for disease-free survival (DFS).

DFS data for 3,971 patients were summarized from 25 studies, including 1,656 patients in the RH group and 2,315 in the RFA group. Patients in the RH group had better DFS than those in RFA group (HR: 0.80, 95% CI: 0.75–0.86, P<0.00001, Z=6.15) (**Figure 4**). Eight studies (23, 25, 33, 38, 45, 46, 52, 55) recorded median DFS with similar results (RH: 13.2–45.4; RFA: 15.2–28.8). Based on 19 relevant studies, DFS was superior in the RH group compared with the RFA group at 1 year (OR: 1.53, 95% CI: 1.30–1.80, P<0.00001, Z=5.18, I2 = 45%), 3 years (OR: 1.47, 95% CI: 1.25–1.71, P<0.00001, Z=4.81, I2 = 0%), and 5 years (OR: 1.64, 95% CI: 1.38–1.95, P<0.00001, Z=5.6, I2 = 28%). The results are shown in bubble plots in **Figure 3B**.

### 3.3 Secondary outcomes

#### 3.3.1 Main postoperative complications

**Figure 5:**
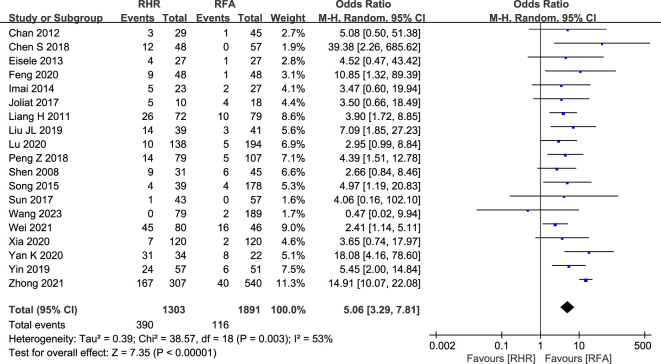
Forest plot for comparison of odds ratios for main postoperative complications.

A total of 19 studies (RH group: 1,303; RFA group: 1,891) reported major postoperative complications (9, 20, 22, 25, 29–32, 34–36, 38, 40, 41, 45, 47, 53, 55, 56). The RFA group had fewer major complications than the RH group (OR: 5.06, 95% CI: 3.29–7.81, P<0.00001, Z=7.35) (**Figure 5**). The heterogeneity between the two groups was too large (I2 = 53%, P=0.003) to be suitable for a fixed-effects model and a random-effects model was required.

#### 3.3.2 Postoperative recurrence rate and mortality

**Figure 6:**
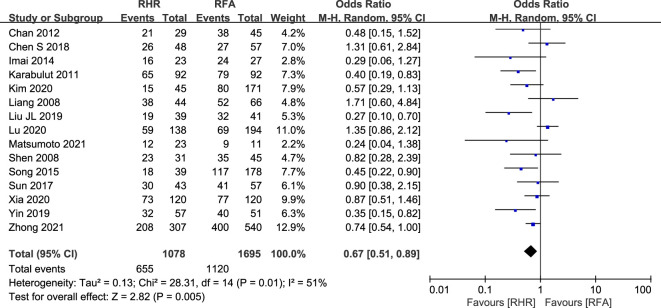
Forest plot for comparison of odds ratios for postoperative recurrence rate.

**Figure 7:**
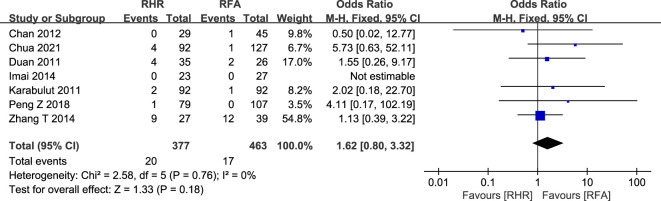
Forest plot for comparison of odds ratios for mortality.

In this study, a total of 15 relevant studies (RH group: 1,078; RFA group: 1,695) reported postoperative recurrence (20, 22, 28, 30, 31, 34, 35, 38, 41, 45, 47, 49, 52–54) (**Figure 6**). The postoperative recurrence rate was lower in the RH group compared with the RFA group (OR: 0.67, 95% CI: 0.51–0.89, P=0.005, Z=2.82). In terms of postoperative mortality (**Figure 7**), we pooled data for 840 patients from seven relevant studies (RH group: 377 patients; RFA group: 463 patients) (20, 23, 24, 32, 43, 47, 54). There was no significant difference between the two groups (OR: 1.62, 95% CI: 0.80–3.32, P=0.18, Z=1.33).

#### 3.3.3 Cost-effectiveness

**Figure 8:**
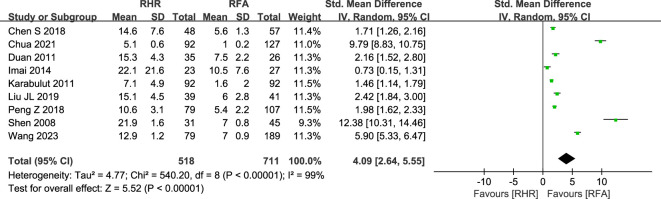
Forest plot for comparison of days of hospitalization.

Considering the patients' economic situations, we analyzed days of hospitalization and the cost of hospitalization. Nine studies (RH group: 518 patients; RFA group: 711 patients) were analyzed in terms of days of hospitalization (**Figure 8**) (22–24, 30, 32, 34, 36, 47, 54) and showed that RFA was superior to RH (SMD: 4.09, 95% CI: 2.64–5.55, P<0.00001, Z=5.52). Hospitalization expenditure was analyzed in two studies: Duan et al (24). (RH group: 26,150.66 ± 7,923.69 yuan; RFA group: 21,135.00 ± 1,156.76 yuan) and Xiao et al (39). (RH group: $7537.48 [6418.66, 8825.05]; RFA group: $3964.15 [2938.22, 4774.56]). There was insufficient data to construct a forest plot for overall analysis, but spending was significantly less in the RFA group compared with the RH group, in accordance with five previous studies (35, 41, 56–58).

### 3.4 Subgroup analysis

We conducted subgroup analyses in terms of tumor size, patient origin, and date of publication (before and after 2015). Patients were divided into two groups based on tumor size (with a 3-cm cutoff). In terms of OS, 16 studies provided data for patients with tumors ≤ 3cm (Additional File 1 **Supplementary Figure S1**) (20, 22, 26, 27, 29–31, 33, 35–38, 44, 46, 49, 53), which showed that the RH group was superior to the RFA group (HR: 0.84, 95% CI: 0.73–0.96, P=0.01, Z=2.53). Three studies included data for patients with tumors >3 cm in diameter (Additional File 1 **Supplementary Figure S1**) (23, 52, 54), but there was no significant difference between the groups (HR: 0.87, 95% CI: 0.72–1.05, P=0.15, Z=1.42) and no heterogeneity between the groups (I2 = 0%, P=0.95). Regarding DFS, 11 studies contained data for patients with tumors ≤3 cm (20, 22, 27, 30, 31, 33, 35, 36, 38, 49, 53), showing that the RH group was superior to the RFA group (HR: 0.75, 95% CI: 0.66–0.85, P<0.00001, Z=4.57), with less heterogeneity between the two groups (I2 = 2%, P=0.42), and a fixed-effects model could be chosen (Additional File 1 **Supplementary Figure S1**). For tumors >3 cm, four relevant studies (23, 40, 52, 54) showed no significant difference (HR: 0.90, 95% CI: 0.63–1.28, P=0.55, Z=0.59), with greater heterogeneity between the two groups (I2 = 65%, P=0.04) (Additional File 1 **Supplementary Figure S1**).

We also divided patients into Chinese- and non-Chinese based on patient origin. In terms of OS (Additional File 1 **Supplementary Figure S2**), there was no significant difference between the two surgical modalities in 23 studies included in the Chinese group (HR: 0.91, 95% CI: 0.83–1.01, P=0.08, Z=1.77), in 12 studies included in the non-Chinese group (HR: 0.92, 95% CI: 0.79–1.08, P=0.33, Z=0.98), or overall, (HR: 0.92, 95% CI: 0.84–1.00, P=0.04, Z =2.02). Regarding DFS (Additional File 1 **Supplementary Figure S2**), the RH group was superior to the RFA group in 16 studies in the Chinese group (HR: 0.80, 95% CI: 0.74–0.87, P<0.0001, Z =5.52) in nine studies in the non-Chinese group (HR: 0.80, 95% CI: 0.69–0.94, P=0.007, Z=2.70), and overall (HR: 0.80, 95% CI: 0.75–-0.86, P<0.00001, Z=6.15). Finally, we compared the groups based on the date of publication. In terms of OS (Additional File 1 **Supplementary Figure S3**), there was no significant difference between the two surgical modalities in 17 studies in the pre-2015 group (HR: 0.93, 95% CI: 0.82–1.07, P=0.31, Z=1.02), in 18 studies in the post-2015 group (HR: 0.91, 95% CI: 0.81–1.01, P=0.08, Z=1.77), or overall (HR: 0.92, 95% CI: 0.84–1.00, P=0.04, Z =2.02). In terms of DFS (Additional File 1 **Supplementary Figure S3**), RH was superior to RFA in the pre-2015 group (HR: 0.78, 95% CI: 0.68–0.89, P=0.0003, Z=3.61), the post-2015 group (HR: 0.81, 95% CI: 0.75–0.88, P<0.0001, Z=5.01), and overall (HR: 0.80, 95% CI: 0.75–0.86, P<0.00001, Z=6.15).

### 3.5 Publication bias assessment

**Figure 9:**
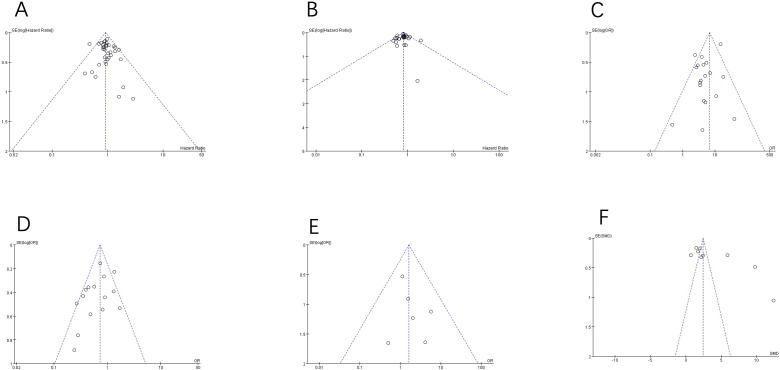
Inverted funnel plot analysis of the primary and secondary outcomes between RH and RFA. **(A)** OS; **(B)** DFS; **(C)** main postoperative complications; **(D)** postoperative recurrence rate; **(E)** mortality; **(F)** days of hospitalization.

The possibility of publication bias was analyzed by the inverted funnel plot. The plot resembles a symmetric inverted funnel (the 95% CI). It is notable that in **Figures 9A–D**, illustrating the inverted funnel plot analyses of OS, DFS, main postoperative complications and postoperative recurrence rate respectively, only one study lay outside the 95% CI axis. In **Figure 9E**, the inverted funnel plot analysis of the postoperative mortality, there was no study outside the 95% CI axis. In **Figure 9F**, the inverted funnel plot analysis of the days of hospitalization, there was a few studies outside the 95% CI axis. We therefore conclude that there is no evidence of publication bias in our analysis.

## 4 Discussion

We conducted a systematic review and meta-analysis of the largest and most up-to-date literature to evaluate the safety and efficacy of RH and RHA in patients with RHCC. This study included two RCTs, which provided a high level of evidence and thus improved the results of the study. The results showed that RH was superior to RFA in terms of long-term prognosis (OS, DFS) in patients with RHCC; however, RFA was superior in terms of the incidence of major postoperative complications and was preferable in terms of the patient's financial situation.

RHCC usually refers to tumors that reappear in the liver or newly developed tumors in the tissues and organs around the liver. The postoperative recurrence rate can be as high as 70% in patients with HCC (3) and the survival rate is only 57.3% (59). Tumor recurrence can be divided into three types according to the location: intrahepatic recurrence, extrahepatic recurrence, and intrahepatic with extrahepatic recurrence, with intrahepatic recurrence accounting for up to 80% (60). The type of recurrence can also be divided according to the origin of the tumor: recurrence (monoclonal origin, true relapsed HCC) and reoccurrence (polyclonal origin, second primary HCC). The former refers to tumors arising from primary intrahepatic tumors, whereas the latter usually refers to neoplastic tumors (61). Nevola et al. suggested that the recurrence mechanism of HCC is related to the time of recurrence and can be classed as early recurrence (≤2 years) or late recurrence (>2 years). Early recurrence accounts for up to 70% of all recurrences, originating from intrahepatic dissemination of the tumor via the portal circulation, while late recurrence is usually caused by *de novo* tumor development (62). In conclusion, tumor recurrence is an important factor affecting the long-term prognosis of patients, and the safety, effectiveness, and radicality of the treatment modality are key to preventing tumor recurrence.

The results of the current and previous meta-analyses suggested that RH was superior to RFA in terms of the long-term prognosis (OS, DFS) of patients with RHCC (6, 10–12), which might be attributed to the ability of RH to achieve complete tumor eradication. Based on anatomical hepatectomy, resection of the primary tumor can also clear some microvascular invasions and micrometastases that are undetectable during the initial treatment, given a margin of dissection ≥1 cm, thus reducing postoperative recurrence in patients with RHCC (63). RH is still the main treatment for RHCC; however, it is necessary to observe surgical contraindications, consider if the patients can tolerate the procedure, and evaluate the surgical risk factors. In order to achieve radical second surgery, the resection margins must be large enough to reduce the risk of recurrence. The margin is thus a key aspect of RH influencing patient prognosis, and it is therefore crucial to consider the margin range during the resection process.

Increasing importance has recently been paid to RFA in clinical practice. This procedure does not require consideration of the margins, and is mainly used in patients who cannot tolerate surgical resection. Compared with RH, RFA has less postoperative blood loss (9, 23, 30, 31, 47, 64, 65), shorter operation time (30, 41, 47, 55), lower operative risk, and faster recovery, which greatly reduce hospitalization days (22–24, 30, 32, 34, 36, 47, 54) and reduce the patient’s financial burden (24, 35, 39, 41). Because of its less-invasive nature, RFA can be used as an alternative treatment in patients with early RHCC. Nevertheless, RFA also has some shortcomings. First, RFA may not be able to completely cure some occult tumors or tumors adjacent to main vessels, thus increasing the risk of tumor recurrence. Second, the intense heating of the main area by RFA may cause tumor cells to scatter around the ablation area (23) or spread to other parts of the liver via the needle path (66, 67).

RH is generally more likely to cause postoperative symptoms such as pulmonary atelectasis, infected fever, bile leakage, or abdominal adhesions due to surgical maneuvers, while sub peritoneal hematoma and pneumothorax are more common in RFA (9). The current study showed that RFA was preferable to RH in terms of major postoperative complications, possibly because of the less-invasive nature of RFA. In the case of open re-excision, abdominal adhesions greatly increase the difficulty of the operation, thus increasing the incidence of postoperative complications and affecting the prognosis. Laparoscopic hepatectomy, as a minimally invasive surgery, may thus be a good alternative treatment, to decrease postoperative complications (25, 30, 35, 41, 53).

Tumor number and diameter are known to be independent risk factors for postoperative survival (68–70). In the present study, there was insufficient data on tumor numbers and we therefore focused on tumor diameter. First, we divided patients into two groups based on tumor diameter, with 3 cm as the cutoff. Both surgical approaches were effective in the ≤3 cm group, in terms of both OS and DFS, but RH was superior to RFA group, while there was no significant difference between the two surgical approaches in the >3 cm group. RFA is generally considered to be a better choice for tumors ≤3 cm in diameter, while RH is more suitable for those >3 cm. Surprisingly, the current analysis drew the opposite conclusion, possibly because of the heterogeneity of the included literature. Second, we divided studies according to publication date, with 2015 as the cutoff, to determine if the two surgical procedures had developed over time. There was no significant difference in OS between the pre-2015 and post-2015 studies, but DFS was better in the RH group compared with the RFA group, both pre-2015 and post-2015. Finally, we also divided patients into two groups (Chinese and non-Chinese) according to their country of origin to determine if the effects of the two treatments differed depending on the country. There was no significant difference between the two groups in terms of OS, but RH was superior to RFA in terms of DFS in both the China and non-China groups. RH may thus play a more important role in the postoperative long-term survival of patients with RHCC.

The prevalence of hepatitis B infection and cirrhosis, and AFP expression were generally high in most patients at baseline, suggesting that the recurrence of HCC may be related to these factors. There is currently insufficient data to draw a definitive conclusion, but it can be affirmed that early prevention and treatment are very important for preventing the recurrence of HCC. Meanwhile, whether RHR or RFA, postoperative combined adjuvant therapy (e.g., targeted drugs, immunotherapy) can greatly reduce the tumor recurrence rate, prolong patient survival, improve the prognosis, and enhance the quality of life.

This study had some limitations. First, most of the included studies were non-clinical trials, and there were only two RCTs. Selection bias was thus unavoidable, and more high-quality RCTs are needed. Second, there were some unavoidable confounding factors which could lead to bias, such as patients having two or more diseases or receiving more than two combination treatments, as well as differences in surgeon proficiency. Third, the subgroup analysis of tumor size included little data for tumors >3 cm, making it difficult to obtain an exact result. Fourth, the data on RHCC staging or typing were not clear enough to carry out a subgroup analysis and provide a better treatment plan for patients. Finally, some studies lack detailed information, as evidenced by the numerous "NR" (not reported) entries in **Table 2**, which limits the clinical applicability of the findings.

## 5 Conclusion

Current evidence suggests that RH is better than RFA in terms of the long-term prognosis in patients with RHCC. In contrast however, RFA is preferable in terms of postoperative complications, as well as the economic burden (days of hospitalization, costs). Considering the prognosis of patients with tumors ≤3 cm in diameter, RH is more effective than RFA; however, RH and RFA can achieve similar treatment effects in patients with tumors >3 cm. Further high-quality studies need to be included in future meta-analyses to allow more definite conclusions.

## Data Availability

The original contributions presented in the study are included in the article/**Supplementary Material**. Further inquiries can be directed to the corresponding authors.

## Supplementary material

Supplementary Figure 1
**(A)** Forest plot for comparison of hazard ratios for OS (diameter ≤3 cm). **(B)** Forest plot for comparison of hazard ratios for OS (diameter >3 cm). **(C)** Forest plot for comparison of hazard ratios for DFS (diameter ≤3 cm). **(D)** Forest plot for comparison of hazard ratios for DFS (diameter >3 cm).

Supplementary Figure 2
**(A)** Forest plot for comparison of hazard ratios for OS according to patient origin. **(B)** Forest plot for comparison of hazard ratios for DFS according to patient origin

Supplementary Figure 3
**(A)** Forest plot for comparison of hazard ratios for OS according to publication date. **(B)** Forest plot for comparison of hazard ratio for DFS according to publication date.

## References

[1] Sung H Ferlay J Siegel RL Laversanne M Soerjomataram I Jemal A . Global cancer statistics 2020: GLOBOCAN estimates of incidence and mortality worldwide for 36 cancers in 185 countries. CA: Cancer J Clin. (2021) 71:209–49. doi: 10.3322/caac.21660 33538338

[2] Ganesan P Kulik LM . Hepatocellular carcinoma: new developments. Clinics liver dis. (2023) 27:85–102. doi: 10.1016/j.cld.2022.08.004 36400469

[3] Clinical Practice Guidelines EASL . Management of hepatocellular carcinoma. J hepatol. (2018) 69:182–236. doi: 10.1016/j.jhep.2018.03.019 29628281

[4] Zheng J Cai J Tao L Kirih MA Shen Z Xu J . Comparison on the efficacy and prognosis of different strategies for intrahepatic recurrent hepatocellular carcinoma: A systematic review and Bayesian network meta-analysis. Int J Surg (London England). (2020) 83:196–204. doi: 10.1016/j.ijsu.2020.09.031 32980518

[5] Cai H Kong W Zhou T Qiu Y . Radiofrequency ablation versus reresection in treating recurrent hepatocellular carcinoma: a meta-analysis. Medicine. (2014) 93:e122. doi: 10.1097/MD.0000000000000122 25396332 PMC4616312

[6] Chen Z Wang J Lin Y . Comparison of the efficacy and safety of repeated hepatectomy and radiofrequency ablation in the treatment of primary recurrent liver cancer: a meta-analysis. World J Surg Oncol. (2022) 20:182. doi: 10.1186/s12957-022-02649-4 35668464 PMC9169306

[7] Gavriilidis P Askari A Azoulay D . Survival following redo hepatectomy vs radiofrequency ablation for recurrent hepatocellular carcinoma: a systematic review and meta-analysis. HPB: Off J Int Hepato Pancreato Biliary Assoc. (2017) 19:3–9. doi: 10.1016/j.hpb.2016.10.003 28341429

[8] Liu J Zhao J Gu HAO Zhu Z . Repeat hepatic resection VS radiofrequency ablation for the treatment of recurrent hepatocellular carcinoma: an updated meta-analysis. Minimal invasive Ther Allied technol: MITAT. (2022) 31:332–41. doi: 10.1080/13645706.2020.1839775 33143517

[9] Wei F Huang Q Zhou Y Luo L Zeng Y . Radiofrequency ablation versus repeat hepatectomy in the treatment of recurrent hepatocellular carcinoma in subcapsular location: a retrospective cohort study. World J Surg Oncol. (2021) 19:175. doi: 10.1186/s12957-021-02277-4 34127007 PMC8204439

[10] Yang D Zhuang B Wang Y Xie X Xie X . Radiofrequency ablation versus hepatic resection for recurrent hepatocellular carcinoma: an updated meta-analysis. BMC gastroenterol. (2020) 20:402. doi: 10.1186/s12876-020-01544-0 33246417 PMC7693504

[11] Yang Y Yu H Tan X You Y Liu F Zhao T . Liver resection versus radiofrequency ablation for recurrent hepatocellular carcinoma: a systematic review and meta-analysis. Int J hyperther. (2021) 38:875–86. doi: 10.1080/02656736.2021.1933218 34078221

[12] Yeow M Zhao JJ Fong KY Wong J Tan AYH Kam JH . Radiofrequency ablation versus repeat hepatectomy for recurrent hepatocellular carcinoma: A systematic review and meta-analysis. World J surge. (2022) 46:2778–87. doi: 10.1007/s00268-022-06691-x 35989371

[13] Yuan BH Zhu YK Zou XM Zhou HD Li RH Zhong JH . Repeat hepatic resection versus percutaneous ablation for the treatment of recurrent hepatocellular carcinoma: meta-analysis. BJS Open. (2022) 6. doi: 10.1093/bjsopen/zrac036 PMC9048940 35482024

[14] Cook DA Reed DA . Appraising the quality of medical education research methods: the Medical Education Research Study Quality Instrument and the Newcastle-Ottawa Scale-Education. Acad Med. (2015) 90:1067–76. doi: 10.1097/ACM.0000000000000786 26107881

[15] Damaskos C Garmpis N Psilopatis I Dimitroulis D . Natural ending or surgical complication: is it the time to reconsider the clavien-dindo classification system? Maedica. (2022) 17:939–47. doi: 10.26574/maedica.2022.17.4.939 PMC9923058 36818261

[16] Cumpston M Li T Page MJ Chandler J Welch VA Higgins JP . Updated guidance for trusted systematic reviews: a new edition of the Cochrane Handbook for Systematic Reviews of Interventions. Cochrane Database system Rev. (2019) 10:Ed000142. doi: 10.1002/14651858.ED000142 31643080 PMC10284251

[17] Tierney JF Stewart LA Ghersi D Burdett S Sydes MR . Practical methods for incorporating summary time-to-event data into meta-analysis. Trials. (2007) 8:16. doi: 10.1186/1745-6215-8-16 17555582 PMC1920534

[18] Brush PL Sherman M Lambrechts MJ . Interpreting meta-analyses: A guide to funnel and forest plots. Clin Spine surge. (2024) 37:40–2. doi: 10.1097/BSD.0000000000001534 37684723

[19] Mavridis D Salanti G . How to assess publication bias: funnel plot, trim-and-fill method and selection models. Evidence-Based Ment Health. (2014) 17:30. doi: 10.1136/eb-2013-101699 PMC13404685 24477535

[20] Chan AC Poon RT Cheung TT Chok KS Chan SC Fan ST . Survival analysis of re-resection versus radiofrequency ablation for intrahepatic recurrence after hepatectomy for hepatocellular carcinoma. World J surge. (2012) 36:151–6. doi: 10.1007/s00268-011-1323-0 PMC3243850 22030561

[21] Chen K Liu XY Teng YX Li MJ Li YS Huang T . Rehepatectomy and radiofrequency ablation for patients with recurrent hepatocellular carcinoma. Zhong Guo Shi Yong Wai Ke Za Zhi. (2019) 39:1060–4.

[22] Chen SL Peng ZW Xiao H Lin MX Chen ZB Jiang CL . Combined radiofrequency ablation and ethanol injection versus repeat hepatectomy for elderly patients with recurrent hepatocellular carcinoma after initial hepatic surgery. Int J Hypertherm. (2018) 34:1029–37. doi: 10.1080/02656736.2017.1387941 28974113

[23] Chua DW Koh YX Syn NL Chuan TY Yao TJ Lee SY . Repeat hepatectomy versus radiofrequency ablation in management of recurrent hepatocellular carcinoma: an average treatment effect analysis. Ann Surg Oncol. (2021) 28:7731–40. doi: 10.1245/s10434-021-09948-2 33969464

[24] Duan JC Yue HY Liu K Wu MC Yang JH . Percutaneous radiofrequency ablation versus repeat hepatectomy for recurrent hepatocellular carcinoma:retrospective randomized control study %J Journal of Medical Colleges of PLA. J Med Colleges PLA. (2011) 26:316–23.

[25] Feng YM Wu H Huang DQ Xu CH Zheng H Maeda M . Radiofrequency ablation versus repeat resection for recurrent hepatocellular carcinoma (≤ 5 cm) after initial curative resection. Eur Radiol. (2020) 30:6357–68. doi: 10.1007/s00330-020-06990-8 32529568

[26] Ho CM Lee PH Shau WY Ho MC Wu YM Hu RH . Survival in patients with recurrent hepatocellular carcinoma after primary hepatectomy: comparative effectiveness of treatment modalities. Surgery. (2012) 151:700–9. doi: 10.1016/j.surg.2011.12.015 22284764

[27] Huang J Yan L Wu H Yang J Liao M Zeng Y . Is radiofrequency ablation applicable for recurrent hepatocellular carcinoma after liver transplantation? J Surg Res. (2016) 200:122–30. doi: 10.1016/j.jss.2015.07.033 26277218

[28] Liang HH Chen MS Peng ZW Zhang YJ Zhang YQ Li JQ . Percutaneous radiofrequency ablation versus repeat hepatectomy for recurrent hepatocellular carcinoma: a retrospective study. Ann Surg Oncol. (2008) 15:3484–93. doi: 10.1245/s10434-008-0076-y 18679754

[29] Liang H Peng Z Chen M . Effects of percutaneous radiofrequency ablation and repeat hepatectomy for the treatment of solitary recurrent hepatocellular carcinoma with the diameter no more than 3 cm. Chin J Dig Surg. (2011) 10:36–9.

[30] Liu JL Huang D Cao L Wang XJ Li JW Chen J . Laparoscopic hepatectomy versus radiofrequency ablation in treatment of recurrent hepatocellular carcinoma: a prospective randomized control study based on interim follow-up analysis. Di San Jun Yi Da Xue Xue Bao. (2019) 41:467–72.

[31] Lu LH Mei J Kan AN Ling YH Li SH Wei W . Treatment optimization for recurrent hepatocellular carcinoma: Repeat hepatic resection versus radiofrequency ablation. Cancer Med. (2020) 9:2997–3005. doi: 10.1002/cam4.2951 32108433 PMC7196061

[32] Peng Z Wei M Chen S Lin M Jiang C Mei J . Combined transcatheter arterial chemoembolization and radiofrequency ablation versus hepatectomy for recurrent hepatocellular carcinoma after initial surgery: a propensity score matching study. Eur Radiol. (2018) 28:3522–31. doi: 10.1007/s00330-017-5166-4 29536241

[33] Ren ZG Gan YH Fan J . Treatment of postoperative recurrence of hepatocellular carcinoma with radiofrequency ablation comparing with repeated surgical resection. Zhong Hua Wai Ke Za Zhi. (2008) 46:1614–6. 19094752

[34] Shen Q Xue HZ Jiang QF . Comparison of the effects of percutaneous radiofrequency ablation and surgical re-resection on postoperative recurrence of hepatocellular carcinoma. Chin J Clin Oncol. (2008) 35:1088–92.

[35] Sun WC Chen IS Liang HL Tsai CC Chen YC Wang BW . Comparison of repeated surgical resection and radiofrequency ablation for small recurrent hepatocellular carcinoma after primary resection. Oncotarget. (2017) 8:104571–81. doi: 10.18632/oncotarget.21604 PMC5732828 29262662

[36] Wang C Li K Huang Z Yuan Y He W Zheng Y . Repeat hepatectomy versus percutaneous ablation for recurrent hepatocellular carcinoma: emphasis on the impact of early or late recurrence. J Cancer Res Clin Oncol. (2023) 149:15113–25. doi: 10.1007/s00432-023-05286-w PMC11797161 37632543

[37] Wang K Liu G Li J Yan Z Xia Y Wan X . Early intrahepatic recurrence of hepatocellular carcinoma after hepatectomy treated with re-hepatectomy, ablation or chemoembolization: a prospective cohort study. Eur J Surg Oncol. (2015) 41:236–42. doi: 10.1016/j.ejso.2014.11.002 25434327

[38] Xia Y Li J Liu GH Wang K Qian GJ Lu ZH . Long-term effects of repeat hepatectomy vs percutaneous radiofrequency ablation among patients with recurrent hepatocellular carcinoma A randomized clinical trial. JAMA Oncol. (2020) 6:255–63. doi: 10.1001/jamaoncol.2019.4477 PMC6902111 31774468

[39] Xiao H Chen ZB Jin HL Li B Xu LX Guo Y . Treatment selection of recurrent hepatocellular carcinoma with microvascular invasion at the initial hepatectomy. Am J Transl Res. (2019) 11:1864–75. PMC6456518 30972210

[40] Yan K Liu W Qin XM Bai GJ . Recurrent small hepatocellular carcinoma; percutaneous radiofrequency ablation; surgical resection. Gan Dan Yi Wai Ke Za Zhi. (2020) 32:286–9.

[41] Yin XL Hua TQ Liang C Chen Z . Efficacy of re-resection versus radiofrequency ablation for recurrent Barcelona Clinic Liver Cancer stage 0/A hepatocellular carcinoma (HCC) after resection for primary HCC. Trans Cancer Res. (2019) 8:1035–45. doi: 10.21037/tcr.2019.06.11 PMC8797533 35116847

[42] Zhang H Xu XB He XJ Liu CL Zhao MY Li WB . Comparison of the efficacy between re-operation and radiofrequency ablation on postoperative recurrent carcinoma. Xi Bu Yi Xue. (2013) 25:1816–8.

[43] Zhang T Li K Luo H Zhang W Zhang L Gao M . Long-term outcomes of percutaneous microwave ablation versus repeat hepatectomy for treatment of late recurrent small hepatocellular carcinoma: a retrospective study. Zhong Hua Yi Xue Za Zhi. (2014) 94:2570–2. 25511485

[44] Zhang XY Li C Wen TF Yan LN Li B Yang JY . Appropriate treatment strategies for intrahepatic recurrence after curative resection of hepatocellular carcinoma initially within the Milan criteria: according to the recurrence pattern. Eur J Gastroenterol Hepatol. (2015) 27:933–40. doi: 10.1097/MEG.0000000000000383 25933127

[45] Zhong JH Xing BC Zhang WG Chan AWH Chong CCN Serenari M . Repeat hepatic resection versus radiofrequency ablation for recurrent hepatocellular carcinoma: Retrospective multicenter study. Br J Surge. (2022) 109:71–8. doi: 10.1093/bjs/znab340 34643677

[46] Hirokawa F Hayashi M Miyamoto Y Asakuma M Shimizu T Komeda K . Appropriate treatment strategy for intrahepatic recurrence after curative hepatectomy for hepatocellular carcinoma. J gastrointest Surg. (2011) 15:1182–7. doi: 10.1007/s11605-011-1484-z 21557020

[47] Imai K Beppu T Chikamoto A Mima K Okabe H Hayashi H . Salvage treatment for local recurrence of hepatocellular carcinoma after local ablation therapy. Hepatol Res. (2014) 44:E335–345. doi: 10.1111/hepr.2014.44.issue-14 24552247

[48] Kawano Y Sasaki A Kai S Endo Y Iwaki K Uchida H . Prognosis of patients with intrahepatic recurrence after hepatic resection for hepatocellular carcinoma: a retrospective study. Eur J Surg Oncol. (2009) 35:174–9. doi: 10.1016/j.ejso.2008.01.027 18325724

[49] Matsumoto M Yanaga K Shiba H Wakiyama S Sakamoto T Futagawa Y . Treatment of intrahepatic recurrence after hepatectomy for hepatocellular carcinoma. Ann Gastroenterol Surg. (2021) 5:538–52. doi: 10.1002/ags3.12449 PMC8316745 34337303

[50] Saito R Amemiya H Hosomura N Kawaida H Maruyama S Shimizu H . Prognostic significance of treatment strategies for the recurrent hepatocellular carcinomas after radical resection. In Vivo. (2020) 34:1265–70. doi: 10.21873/invivo.11900 PMC7279801 32354917

[51] Umeda Y Matsuda H Sadamori H Matsukawa H Yagi T Fujiwara T . A prognostic model and treatment strategy for intrahepatic recurrence of hepatocellular carcinoma after curative resection. World J surge. (2011) 35:170–7. doi: 10.1007/s00268-010-0794-8 20922387

[52] Kim JM Joh JW Yi NJ Choi GS Kwon CHD Lee KW . Living donor liver transplantation should be cautiously considered as initial treatment in recurrent hepatocellular carcinoma within the Milan criteria after curative liver resection. Ann Transl Med. (2020) 8:288. doi: 10.21037/atm.2020.02.170 32355732 PMC7186733

[53] Song KD Lim HK Rhim H Lee MW Kim YS Lee WJ . Repeated hepatic resection versus radiofrequency ablation for recurrent hepatocellular carcinoma after hepatic resection: A propensity score matching study. Radiology. (2015) 275:599–608. doi: 10.1148/radiol.14141568 25559235

[54] Karabulut K Aucejo F Akyildiz HY Siperstein A Berber E . Resection and radiofrequency ablation in the treatment of hepatocellular carcinoma: a single-center experience. Surg Endosc. (2012) 26:990–7. doi: 10.1007/s00464-011-1983-8 22038164

[55] Eisele RM Chopra SS Lock JF Glanemann M . Treatment of recurrent hepatocellular carcinoma confined to the liver with repeated resection and radiofrequency ablation: a single center experience. Technol Health Care. (2013) 21:9–18. doi: 10.3233/THC-120705 23358055

[56] Joliat GR Allemann P Labgaa I Demartines N Halkic N . Treatment and outcomes of recurrent hepatocellular carcinomas. Langenbeck's Arch surge. (2017) 402:737–44. doi: 10.1007/s00423-017-1582-9 28497194

[57] Cucchetti A Piscaglia F Cescon M Colecchia A Ercolani G Bolondi L . Cost-effectiveness of hepatic resection versus percutaneous radiofrequency ablation for early hepatocellular carcinoma. J hepatol. (2013) 59:300–7. doi: 10.1016/j.jhep.2013.04.009 23603669

[58] Guo WX Sun JX Cheng YQ Shi J Li N Xue J . Percutaneous radiofrequency ablation versus partial hepatectomy for small centrally located hepatocellular carcinoma. World J surge. (2013) 37:602–7. doi: 10.1007/s00268-012-1870-z 23212793

[59] Ren L ZY Zhang M . Rational choice of treatment modality for recurrent hepatocellular carcinoma. J Hepatobil Surge. (2022) 30:317–20.

[60] Tung-Ping-Poon R Fan ST Wong J . Risk factors, prevention, and management of postoperative recurrence after resection of hepatocellular carcinoma. Ann surge. (2000) 232:10–24. doi: 10.1097/00000658-200007000-00003 PMC1421103 10862190

[61] Wang Z ZW . Several focal issues in the diagnosis and treatment of recurrent hepatocellular carcinoma. Chin J Pract Surge. (2019) 39:1027–30.

[62] Nevola R Ruocco R Criscuolo L Villani A Alfano M Beccia D . Predictors of early and late hepatocellular carcinoma recurrence. World J gastroenterol. (2023) 29:1243–60. doi: 10.3748/wjg.v29.i8.1243 PMC10011963 36925456

[63] Shi M Guo RP Lin XJ Zhang YQ Chen MS Zhang CQ . Partial hepatectomy with wide versus narrow resection margin for solitary hepatocellular carcinoma: a prospective randomized trial. Ann Surg. (2007) 245:36–43. doi: 10.1097/01.sla.0000231758.07868.71 17197963 PMC1867934

[64] Lau WY Lai EC . The current role of radiofrequency ablation in the management of hepatocellular carcinoma: a systematic review. Ann surge. (2009) 249:20–5. doi: 10.1097/SLA.0b013e31818eec29 19106671

[65] Omata M Lesmana LA Tateishi R Chen PJ Lin SM Yoshida H . Asian Pacific Association for the Study of the Liver consensus recommendations on hepatocellular carcinoma. Hepatol Int. (2010) 4:439–74. doi: 10.1007/s12072-010-9165-7 PMC2900561 20827404

[66] Masuda T Beppu T Ishiko T Horino K Baba Y Mizumoto T . Intrahepatic dissemination of hepatocellular carcinoma after local ablation therapy. J hepato-biliary-pancreatic surge. (2008) 15:589–95. doi: 10.1007/s00534-007-1288-4 18987928

[67] Stigliano R Marelli L Yu D Davies N Patch D Burroughs AK . Seeding following percutaneous diagnostic and therapeutic approaches for hepatocellular carcinoma. What is the risk and the outcome? Seeding risk for percutaneous approach of HCC. Cancer Treat Rev. (2007) 33:437–47. doi: 10.1016/j.ctrv.2007.04.001 17512669

[68] Lee CW Yu MC Lin G Chiu JC Chiang MH Sung CM . Serum metabolites may be useful markers to assess vascular invasion and identify normal alpha-fetoprotein in hepatocellular carcinoma undergoing liver resection: a pilot study. World J Surg Oncol. (2020) 18:121. doi: 10.1186/s12957-020-01885-w 32493393 PMC7271504

[69] Peng W Li C Zhang X Wen T Chen Z . The impact of thrombocytopenia on prognosis of HBV-related small hepatocellular carcinoma: a propensity score matching analysis. World J Surg Oncol. (2021) 19:46. doi: 10.1186/s12957-021-02160-2 33573630 PMC7879633

[70] Shimada S Kamiyama T Orimo T Nagatsu A Asahi Y Sakamoto Y . Prognoses, outcomes, and clinicopathological characteristics of very elderly patients with hepatocellular carcinoma who underwent hepatectomy. World J Surg Oncol. (2020) 18:122. doi: 10.1186/s12957-020-01899-4 32522259 PMC7288547

