# Supplementary figures and images for: Efficacy of repeat hepatectomy versus radiofrequency ablation for recurrent hepatocellular carcinoma: a Systematic Review and meta-analysis
Source: Front Oncol. 2025 Mar 26;15:1559491. doi: 10.3389/fonc.2025.1559491 (PMC11979987; doi:10.3389/fonc.2025.1559491)

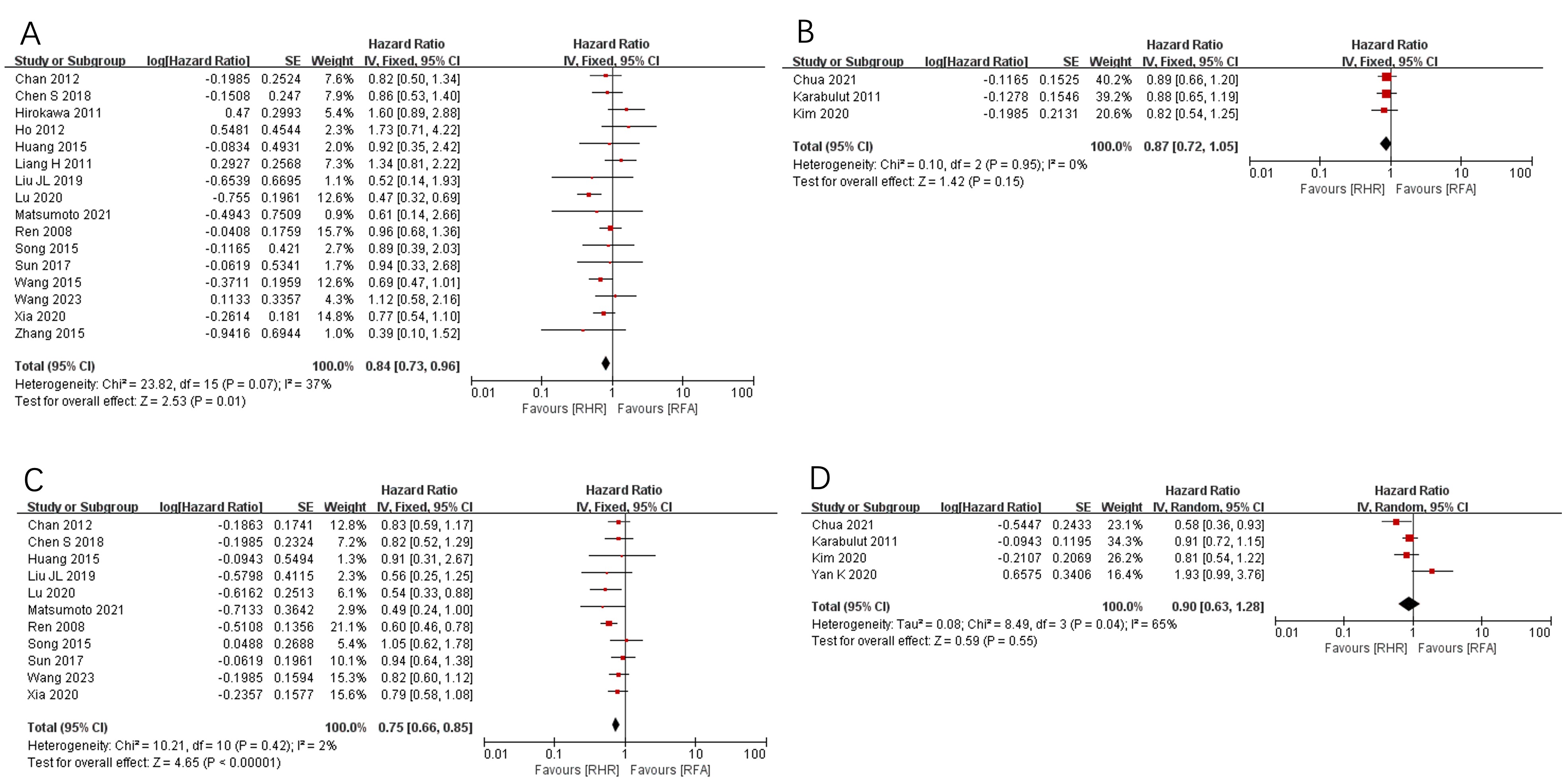

Supplement: Supplementary Figure 1 — (A) Forest plot for comparison of hazard ratios for OS (diameter ≤3 cm). (B) Forest plot for comparison of hazard ratios for OS (diameter >3 cm). (C) Forest plot for comparison of hazard ratios for DFS (diameter ≤3 cm). (D) Forest plot for comparison of hazard ratios for DFS (diameter >3 cm). [file Image1.jpeg]

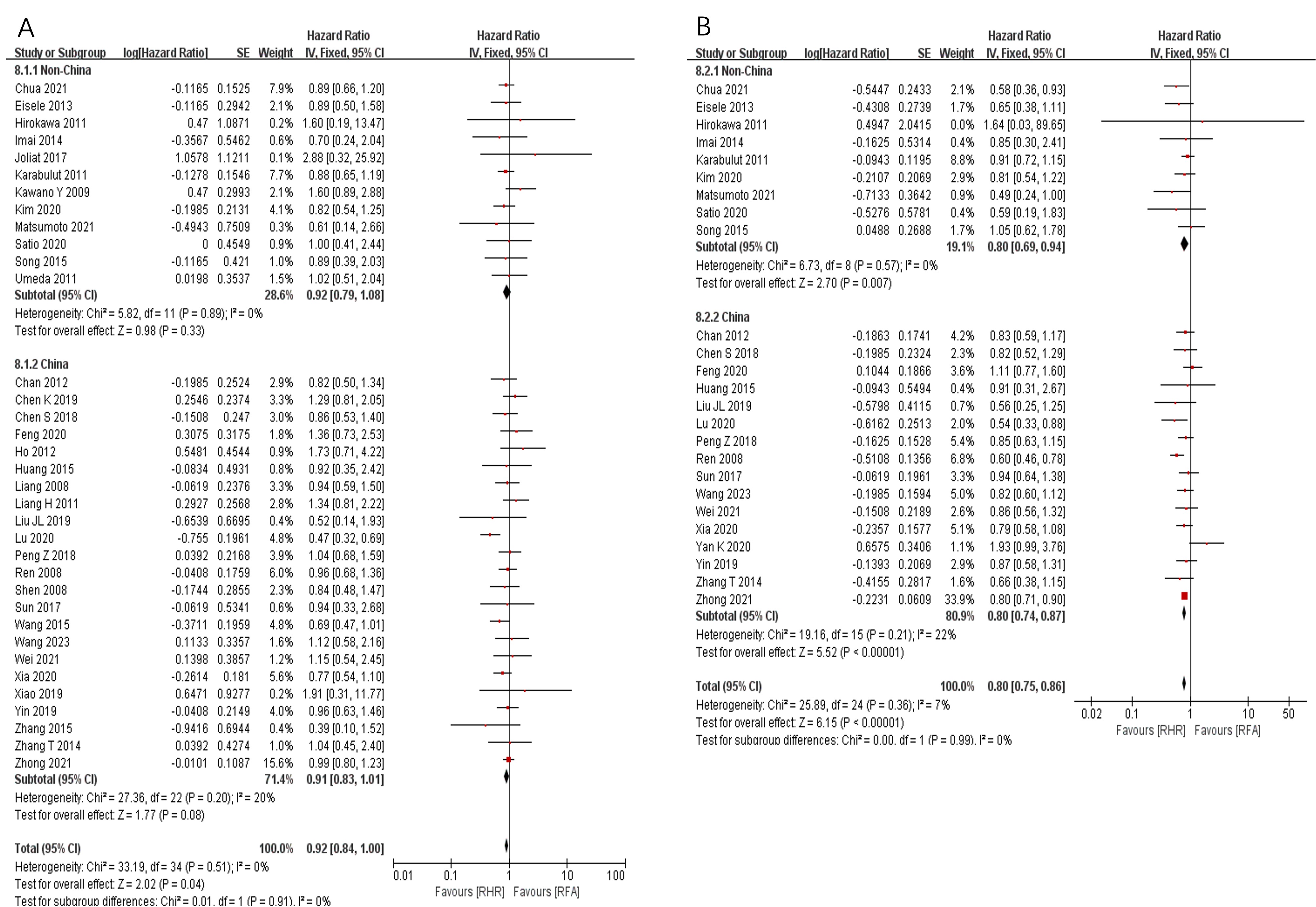

Supplement: Supplementary Figure 2 — (A) Forest plot for comparison of hazard ratios for OS according to patient origin. (B) Forest plot for comparison of hazard ratios for DFS according to patient origin [file Image2.jpeg]

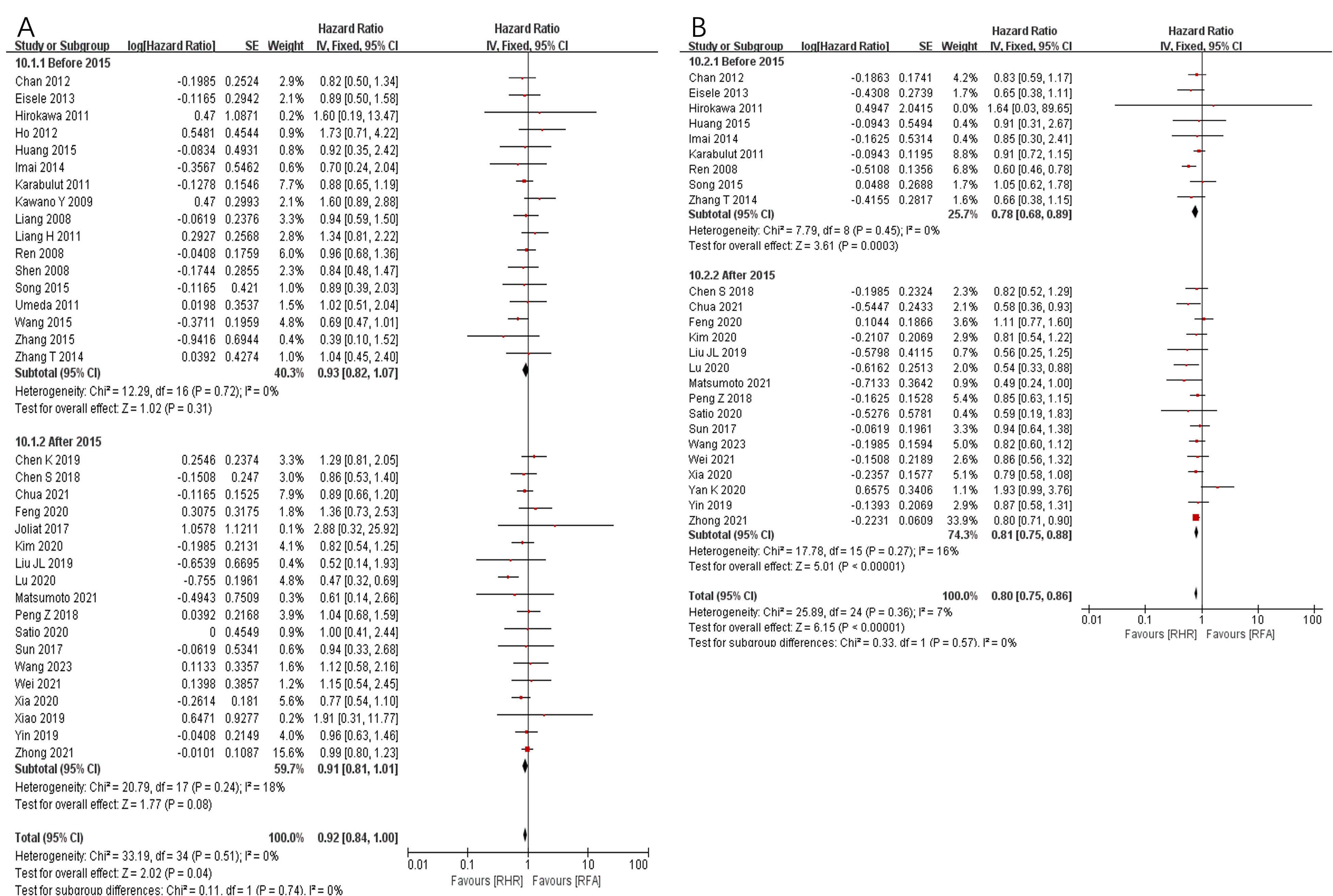

Supplement: Supplementary Figure 3 — (A) Forest plot for comparison of hazard ratios for OS according to publication date. (B) Forest plot for comparison of hazard ratio for DFS according to publication date. [file Image3.jpeg]
